# Effect of adding fenofibrate versus curcumin to glimepiride in patients with type 2 diabetes: a randomized controlled trial

**DOI:** 10.1186/s40360-025-00950-y

**Published:** 2025-06-06

**Authors:** Eman M. Nada, Nashwa M. El-Gharbawy, Haidy Abbas, Rehab H. Werida

**Affiliations:** 1 Department of Clinical Pharmacy, Ministry of Health and Population, Damanhour, Egypt; 2 Department of Internal Medicine (Diabetes & Endocrinology Unit), Faculty of Medicine, Tanta University, Tanta, Egypt; 3 Department of Pharmaceutics, Faculty of Pharmacy, Damanhour University, 22514 Damanhour, Egypt; 4 Department of Clinical Pharmacy & Pharmacy Practice, Faculty of Pharmacy, Damanhour University, Damanhour, 22514 Egypt

**Keywords:** Type 2 diabetes, Curcumin, Fenofibrate, Hs-CRP, Fetuin-A, Sirtuin 1

## Abstract

**Background:**

Type 2 diabetes is a recognized risk factor for the development of cardiovascular disease. Fenofibrate and curcumin were found to be effective in improving hyperlipidemia in patients with diabetes. This study aimed to evaluate the effect of adding fenofibrate versus curcumin on weight, glycemic status, lipids profile, high-sensitivity C-reactive protein (hs-CRP), fetuin-A, and sirtuin 1 in patients with type 2 diabetes treated with glimepiride.

**Method:**

In a double-blind, randomized control trial, 60 patients with type 2 diabetes mellitus were randomly assigned into three groups: Group I was given placebo; Group II curcumin 1100 mg; Group III fenofibrate 160 mg (each administered orally once daily) to ongoing glimepiride 4 mg therapy administered orally once daily for three months. Inclusion criteria were as follows: patients aged 35–70 years, those with uncontrolled type 2 diabetes, hyperlipidemia, and those treated with glimepiride 4 mg. Exclusion criteria were as follows: other types of diabetes, pregnancy, abnormal liver or kidney function tests, using other anti-diabetes medications, and non-adherence to medications. At baseline and after three months of intervention, anthropometric measurements were measured, and blood samples were collected for biochemical analysis of blood glucose, glycated hemoglobin (HbA1c), lipid profile, hs-CRP, fetuin-A, and sirtuin 1. Paired *t*-test and one-way ANOVA were used for normally distributed data. However, the Wilcoxon signed-rank and the Kruskal–Wallis tests were used to analyze non-normally distributed data.

**Results:**

Three months after the intervention, when the three groups were compared, no significant differences were found regarding weight, body mass index, fasting blood glucose, two-hour postprandial glucose (2 h-PPG), and HbA1c (p > 0.05). Compared to placebo, significant decreases were observed in total cholesterol (TC), triglycerides (TG), low-density lipoprotein cholesterol (LDL-C), non-high-density lipoprotein cholesterol (non-HDL-C), very-low-density lipoprotein cholesterol (VLDL-C), coronary risk index (CRI), atherogenic index (AI), and hs-CRP and increasing sirtuin 1 in the fenofibrate (p < 0.001) and curcumin (p < 0.05) groups. High-density lipoprotein cholesterol (HDL-C) levels in the fenofibrate group were found to be significantly higher than in the placebo group (p < 0.001). Furthermore, when compared to curcumin, fenofibrate significantly reduced waist circumferences and fetuin-A and increased sirtuin 1 (p < 0.05).

**Conclusion:**

Both fenofibrate and curcumin are effective at decreasing lipid profiles and improving inflammatory markers in patients with type 2 diabetes. Fenofibrate might have a superior effect in reducing waist circumference, decreasing fetuin-A, and increasing sirtuin 1.

**Trial registration:**

This trial was registered (August 27, 2020) on clinical trial.gov with an identification code NCT04528212. https://www.clinicaltrials.gov/study/NCT04528212.

**Graphical Abstract:**

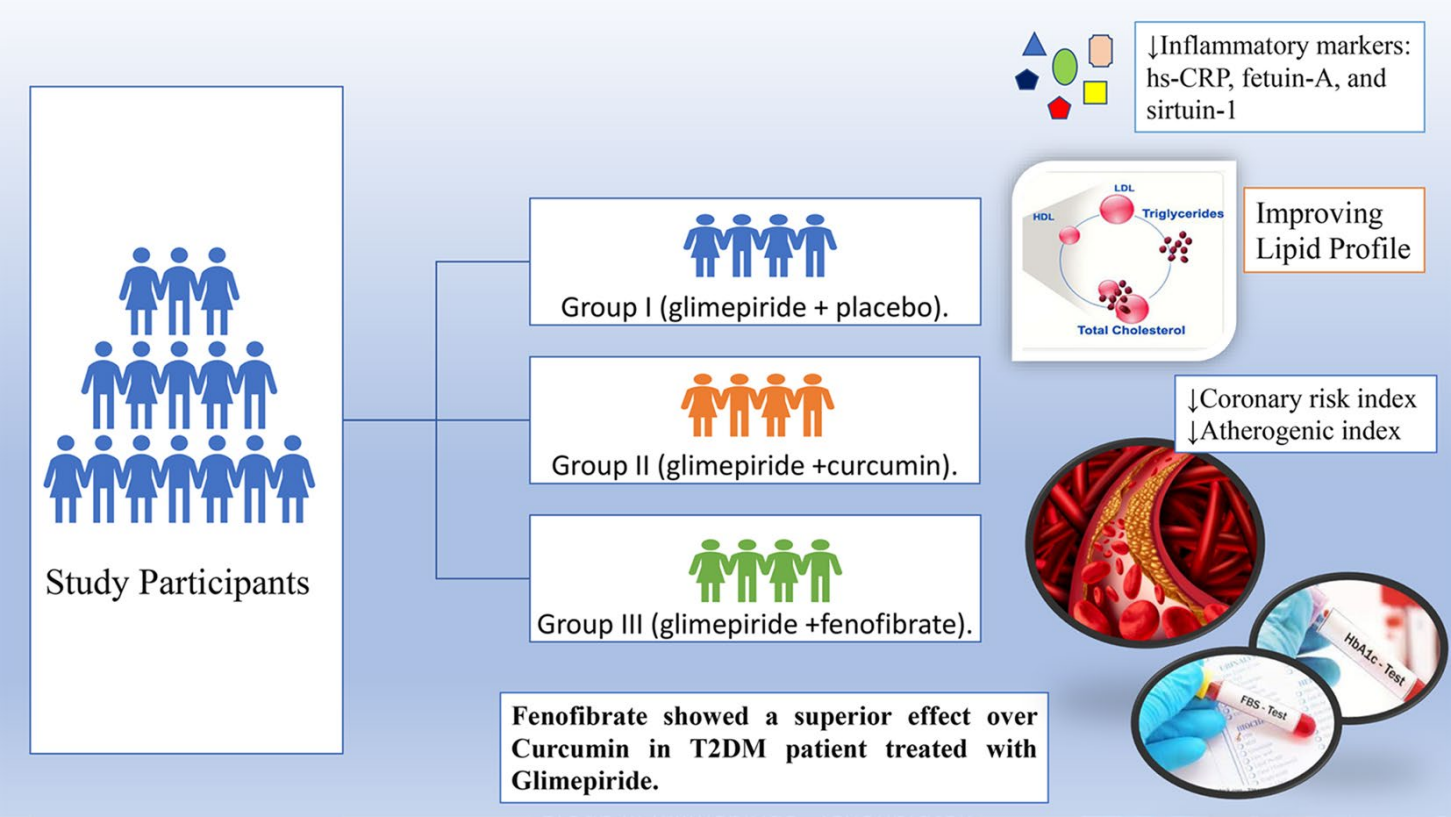

## Introduction

Diabetes has been a significant public health problem, affecting approximately 537 million people worldwide in 2021, and it is expected to reach 783 million by 2045 [1]. Type 2 diabetes mellitus (T2DM) is the most predominant type of diabetes, and it is expected to rise from 6059 individuals per 100,000 in 2017 to 7079 individuals per 100,000 by 2030, indicating a significant increase in cases throughout the world [2].

Dyslipidemia is a significant risk factor for cardiovascular diseases in patients with T2DM. It is associated with a disturbance in lipid metabolism and insulin resistance; therefore, the management of dyslipidemia as a modifiable cardiovascular disease (CVD) risk factor is an essential step in decreasing mortality and preventing coronary heart diseases [3]. The prevalence of cardiovascular complications in T2DM remains a significant challenge, despite the enormous investments in diabetes prevention and management [4]. According to the American Diabetes Association, CVD complications associated with diabetes are responsible for the high percentage of cost in treating diabetes and CVD complications increase mortality and disability in patients with diabetes [5]. Thus, the management of dyslipidemia and CVD in diabetes not only increases the quality of life and productivity but also decreases the economic burden of diabetes on health systems worldwide [6].

Sulfonylureas are widely used as insulin secretagogues in the treatment of T2DM. Glimepiride is classified as a third-generation sulfonylurea, which is approved by the FDA for the management of type 2 diabetes as a monotherapy and in combination therapy with metformin or insulin [7].

According to a study published by Harmer et al., fenofibrate showed significant improvements in lipid profile in adults with type 2 diabetes by decreasing total cholesterol (TC), low-density lipoprotein cholesterol (LDL-C), and triglycerides (TG) and increasing high-density lipoprotein cholesterol (HDL-C) [8]. FIELD trial reported positive results for fenofibrate against microvascular complications of T2DM without knowing the exact mechanism [9].

Curcumin is an active molecule found in the *Curcuma longa* (turmeric) plant’s rhizome [4]. It has many pharmacological actions that have been demonstrated through research [10]. Curcumin may effectively decrease diabetes complications and progression because it has antioxidant effects and prevents free radical formation [11]. The efficacy of curcumin in improving hyperlipidemia and atherosclerosis has been demonstrated through many experimental and clinical studies [12–14].

Through many studies done on diabetic mice, curcumin was effective in reducing the pathogenic processes associated with diabetes and controlling lipid metabolism [15]. In a systematic review done on 16 articles, curcumin supplementation was significant in reducing both fasting blood glucose and glycated hemoglobin and improving the lipid profile [16]. Another study indicated that curcumin might reduce complications of diabetes by decreasing levels of triglycerides and inflammatory markers [17].

Inflammatory marker levels can predict cardiovascular complications in adults with diabetes, as elevated levels of inflammatory markers are linked to cardiovascular mortality and morbidity [18]. C-reactive protein (CRP) is produced by the liver. It is an acute-phase reactant, and its production is associated with the formation of proinflammatory cytokines [19]. It can also be used as a prognostic marker in patients with diabetes to detect potential cardiovascular events [20].

Fetuin-A (α-Heremans–Schmid glycoprotein), produced mainly by the liver and secreted into serum with high concentration, is a physiological inhibitor of insulin receptor tyrosine kinase. Thus, it is associated with insulin resistance, obesity, and a higher risk for T2DM [21]. High circulating plasma levels of fetuin-A are associated with the development of cardiovascular diseases, myocardial infarction, and ischemic stroke [22]. It may be used as a diagnostic marker for the diagnosis of liver and cardiovascular dysfunction in addition to metabolic syndrome-related disorders [23].

On the other hand, sirtuin 1 is a member of the family NAD^+^-dependent enzymes and play a critical role in metabolic process regulation, such as energy homeostasis and the metabolism of adipose tissue [24]. Several inflammatory conditions could be prevented through the upregulation of sirtuin 1 [25]. Sirtuin 1 plays a crucial role in preserving cardiovascular homeostasis and can affect cardiac and endothelial cells through systemic regulation [26]. SIRT1 protects against myocardial infarction, atherothrombosis, and endothelial dysfunction [27].

The above-mentioned-data and based on the scientific importance of curcumin and fenofibrate that has been demonstrated through many studies, that encouraged us to evaluate their potential effects on glycemic control, improving lipid profile, and inflammation, hence decreasing CVD complications. In this context, the main objectives of the present study were to examine the impact of adding fenofibrate versus curcumin on body weight, glycemic status, lipid profile, hs-CRP, fetuin-A, and sirtuin 1 and to assess the association between fetuin-A and sirtuin 1 in patients with type 2 diabetes treated with glimepiride.

## Methods

### Subjects and methods

#### Study design

**Fig. 1:**
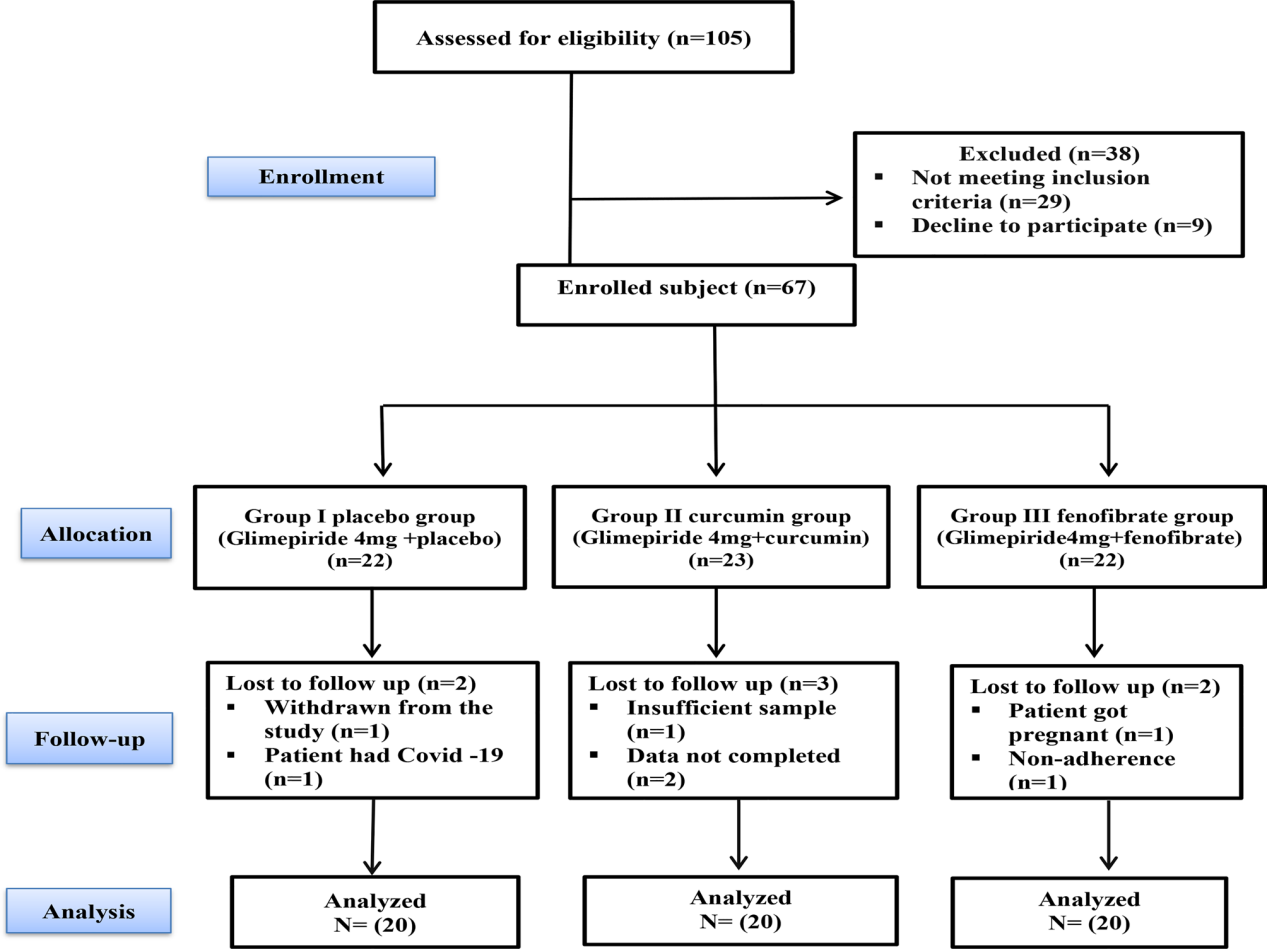
Consort flow diagram for participants’ enrollment, allocation, and follow-up

The study was carried out as a prospective, parallel, double-blind, randomized, placebo-controlled trial. The flowchart of the study protocol is presented in Fig. 1. Patients were recruited from the outpatient clinic of the Diabetes and Endocrinology Unit, Department of Internal Medicine, Tanta University Hospital (Tanta, Egypt). Written informed consent was taken from the patients. Ethical approval for the trial was obtained from the Institutional Research Ethics Committee. Furthermore, this study has been performed following the ethical standards laid down in the 1964 Declaration of Helsinki and its later amendments or comparable ethical standards. Ethical approval for the trial was obtained from the Research Ethics Committee, Faculty of pharmacy, Damanhour University, Damanhour, Egypt (with code no.420PP24).

#### Sample size

The required sample size was calculated using G*Power software version 3.1.0 (Institut für Experimentelle Psychologie, Heinrich Heine Universitat, Dusseldorf, Germany). It was estimated that a total sample size of 60 patients would have a power of 95% to detect a medium to a large effect size of 0.69 in the outcome measure.

#### Inclusion and exclusion criteria

Inclusion criteria were as follows: patients with uncontrolled T2DM, those aged 35–70 years, an HbA1c level of ≥ 7%, an initial plasma level of total cholesterol of 3 · 0–6 · 5 mmol/L (116–251 mg /dl) plus a ratio of TC/HDL-C equal to 4 · 0 or more, or a plasma TG level between 1 · 0–5 · 0 mmol/L (89–443 mg/dl), and those treated with an oral antidiabetics (glimepiride 4 mg) [7].

Exclusion criteria were as follows: other types of DM, pregnancy, lactation or child-bearing potential, abnormal liver function tests, patients with renal impairment (eGFR ≤ 60 ml/min), hypersensitivity to the drug, addition of any anti-diabetes medications or insulin during follow-ups, the use of antioxidants or multivitamins in the last three months, and if the percentage of consumption of medications is less than 90%.

#### Study population

Out of the 105 patients with type 2 diabetes who were screened for their eligibility, 67 patients were enrolled in the study. Diabetes diagnosis was based on medical records and diagnosis can be made with any of the following criteria: FPG ≥ 126 mg/dL and/or 2 h-PPG ≥ 200 mg/dL or HbA1c ≥ 6.5% [28]. Eligible patients were randomly assigned in a double-blind manner by a permuted block randomization method with a computer-generated random sequence in a 1:1:1 ratio to receive either an oral placebo, curcumin, or fenofibrate. The researchers and the participants had no idea about the type of supplementation (placebo, curcumin, or fenofibrate) and the distribution of participants in the groups. Curcumin tablets and fenofibrate tablets were packaged similarly to placebo tablets. All supplements were kept in closed, matched white containers labeled A, B, and C and they were supplied by an independent person. We asked the patients to open the containers at home. The patients were outpatients and they did not know each other, and each patient was met individually at different appointments in follow-up visits.

We told patients not to change their diet, physical activities, and medications throughout the study to avoid any change in the metabolism of glucose and lipids. Patients were randomly allocated into three groups: the first group (n =  22) included patients who received glimepiride 4 mg and a single-dose placebo, the second group (n =  23) comprised patients who received glimepiride 4 mg and single-dose curcumin (1100 mg curcumin with 5 mg black pepper) Pureclinica®, and the third group (n =  22) included patients who received glimepiride 4 mg and single-dose micronized fenofibrate 160 mg Abbott Laboratories®. The duration of the study was 12 weeks.

#### Follow-up

Patients were monitored to control their use of medications every 10 days by phone calls, and scheduled visits were made every 15 days. Successful adherence was established on 90–100% of pill counts. Patients were monitored for the occurrence of any side effects throughout the entire period of the study.

#### Measurement of anthropometric parameters

Measurement of height, weight, and waist circumference of the patients were done before and after the end of the study period in suitable and standard conditions and body mass index (BMI) was calculated as follows: BMI = weight (in kilograms)/squared height (in meters) [29].

#### Measurement of biochemical parameters

A sample of 10 ml blood was collected from each patient after 12 hr overnight fasting at the beginning and the end of the study. The blood samples were centrifuged at 4000 rpm for 10 min. Blood glucose was measured by the glucose oxidase method (Spinreact, Spain), and glycated hemoglobin was measured by the ion exchange method (Biosystems, Spain). The remaining amount of serum sample was divided into two portions: the first portion was used for measurement of lipid profile and the remaining portion was frozen and stored at ˗80 °C until assaying for inflammatory markers.

Serum TC and serum TG were determined by the enzymatic colorimetric method, and serum HDL-C was determined using the precipitation method (Spinreact, Spain). Serum levels of LDL-C, VLDL-C, and non-HDL were calculated using Friedewald’s formula (FF) [30]: LDL-C = (TC) ˗ (HDL-C) ˗ (TG/5), VLDL-C=TG/5, and non-HDL = TC ˗ HDL-C. Moreover, coronary risk index (CRI =  TC/HDL-C) and atherogenic index (AI =  LDL-C/HDL-C) were calculated. Then, serum hs-CRP levels were measured by immunoturbidimetry (Cobas®, Roche diagnostics GmbH), while fetuin-A and sirtuin 1 levels (SIRT1) were evaluated using ELISA kits (Sunred Biotechnology Company®, Shanghai, China). After the sample was collected, the patients were asked to drink a liquid that contained around 75 gm of glucose to measure 2 h-PPG glucose test after two hours of drinking.

#### Study endpoints

The primary efficacy endpoint was the change from baseline in fasting blood glucose, 2 h-PPG glucose levels, and glycated hemoglobin after three months. Key secondary endpoints included the change in inflammatory markers and lipid profile after three months of intervention. Additional secondary endpoints included the change in the measurements of weight, BMI, and waist circumference after three months of intervention. Safety endpoints and tolerability were evaluated throughout the assessment of hypoglycemic episodes, the incidence of adverse events (AEs), and discontinuations due to side effects, in addition to findings from physical examinations and clinical laboratory evaluations.

#### Statistical analyses

The data collected were analyzed using software statistical computer package SPSS version 22.0 (SPSS Inc, Chicago, IL, USA). Kolmogorov–Smirnov and Shapiro–Wilk normality tests were used to assess the normality of the studied parameters. Normally distributed variables were presented as mean ± standard deviation and were analyzed using paired *t*-test to compare before and after the intervention, and one-way ANOVA (Tukey’s HSD post hoc test) was used to compare the three groups. Variables that do not follow normal distribution were expressed as median (interquartile range: 25–75) and were analyzed using Wilcoxon signed-rank test to compare before and after the intervention and the Kruskal–Wallis test to compare between the three studied groups. Categorical variables were expressed as number or percentage and were analyzed using the chi-square post hoc test to compare between the three groups. Association between measured variables was performed using the Pearson’s correlation coefficient. Differences were considered significant at p < 0.05.

## Results

The screening, enrollment, and follow-up process of the study subjects are demonstrated in Fig. 1. Of 105 patients, nine were not interested and declined to join the study. In this context, 96 patients attended the screening, and 29 were excluded from the study because they did not meet the inclusion criteria. Therefore, the remaining 67 patients were randomized into three groups: 22 patients in Group I (glimepiride 4 mg and placebo), 23 patients in Group II (glimepiride 4 mg and curcumin 1100 mg), and 22 patients in Group III (glimepiride 4 mg and fenofibrate 160 mg).

During the follow-up period, two patients dropped out from Group I, one patient withdrew from the study, and the other patient had COVID-19 and was admitted to the intensive care unit, so he did not complete the study. In Group II, three patients dropped out, one patient did not have sufficient samples, and the remaining two patients did not have completed data. In Group III, two patients dropped out, one patient got pregnant, and the other patient did not adhere to the medications. The mean age of all study participants was 56.62 ± 6.59 years old, the mean duration of having type 2 diabetes was 6.9 ± 3.56 years, and 61.7% of the enrolled patients were females. Hypertension was the most common documented medical history among participants (46.67%).

Table 1 shows the baseline data of the study participants before the intervention. At baseline, there was a nonsignificant difference between the three groups regarding the age, gender, duration of diabetes, weight, height, BMI, FBG, 2 h-PPG, HbA1c%, TC, TG, HDL-C, LDL-C, non-HDL-C, VLDL-C, CRI, AI, and the inflammatory markers (hs-CRP, fetuin-A, and sirtuin 1) (p > 0.05).

**Table 1:** Basal Characteristics of all enrolled patients

Variables	Group I (n = 20) placebo group	Group II (n = 20) curcumin group	Group III (n = 20) Fenofibrate group	P*	P_1_	P_2_	P_3_
Age of patient (years)	56.15 ± 5.02	57.40 ± 6.48	56.30 ± 8.16	0.81	0.86	1.00	0.83
Gender (M/F)	8/12	9 /11	6 /14	0.61	0.64	0.76	0.98
Duration of Diabetes (years)	6.60 ± 2.98	7.60 ± 4.33	6.50 ± 3.30	0.57	0.60	1.00	0.65
Hypertension (yes /no)	11 /9	8/12	9/11	0.63	0.98	0.77	0.66
Weight (kg)	84.55 ± 10.78	93.70 ± 16.48	92.15 ± 14.79	0.10	0.94	0.22	0.11
Height (m)	1.64 ± 0.06	1.66 ± 0.05	1.63 ± 0.07	0.13	0.12	0.82	0.36
BMI (kg/m2)	32.00 (28.00–35.75)	32.00(30.25–38.50)	33.50 (31.00–37.75)	0.24	0.62	0.11	0.23
waist (cm)	115.75 ± 10.40	123.20 ± 11.15	116.80 ± 10.70	0.07	0.15	0.95	0.08
FBG (mg/dl)	146.80 ± 26.80	160.15 ± 39.26	148.50 ± 24.73	0.34	0.46	0.98	0.37
2 h-PPG (mg/dl)	218.30 ± 45.61	224.70 ± 48.18	220.25 ± 40.85	0.90	0.95	0.99	0.90
HbA1c%	8.04 ± 0.57	8.36 ± 0.86	8.24 ± 0.63	0.35	0.85	0.64	0.32
TC (mg/dl)	222.65 ± 44.63	226.85 ± 38.36	217.85 ± 38.11	0.78	0.76	0.93	0.94
TG (mg /dl)	158.05 ± 46.47	174.10 ± 51.72	192.20 ± 39.05	0.07	0.43	0.06	0.52
HDL-C (mg/dl)	44.75 ± 6.07	44.8 ± 7.33	39.65 ± 8.85	0.05	0.09	0.09	0.98
LDL-C (mg /dl)	146.30 ± 41.86	147.20 ± 41.65	139.70 ± 35.37	0.81	0.82	0.86	1.00
Non-HDL-C (mg /dl)	177.90 ± 43.88	182.05 ± 40.28	178.20 ± 34.25	0.93	0.95	0.98	0.94
VLDL-C (mg/dl)	31.60 ± 9.34	34.85 ± 10.32	38.50 ± 7.86	0.07	0.43	0.06	0.51
CRI (TC/ HDL)	4.95(3.95–6.00)	5.05(4.15–6.03)	5.45)4.90–5.95)	0.25	0.20	0.12	0.79
AI (LDL/ HDL)	3.35(2.43–4.38)	3.20 (2.30–4.03)	3.50 (2.70–3.95)	0.63	0.36	0.47	0.85
hs-CRP (mg /L)	9.39 ± 5.08	10.51 ± 4.82	11.50 ± 5.49	0.44	0.82	0.40	0.77
Fetuin-A (mg /L)	132.78 ± 38.61	140.56 ± 43.15	157.03 ± 66.49	0.31	0.57	0.30	0.88
Sirtuin 1 (ng/ml)	3.40 ± 0.93	3.10 ± 0.84	2.74 ± 1.02	0.09	0.44	0.07	0.57

*Abbreviations* - M/F: male/female ratio, BMI: body mass index, FBG: fasting blood glucose, 2 h-PPG: two-hour postprandial glucose, HbA1c: glycated hemoglobin, TC: Total Cholesterol, HDL-C: high-density lipoprotein cholesterol, LDL-C: low-density lipoprotein cholesterol, VLDL- C: very low-density lipoprotein cholesterol, TG: Triglycerides, CRI: Coronary Risk Index, AI: Atherogenic index, hs-CRP: high sensitivity C- reactive protein; Values are presented as mean ± SD for variables with normal distribution, median and interquartile range for non-normal distributed variables. One way Anova followed by post hock Tukey’s HDS test was performed for parametric data or the Kruskal-Wallis test for non-parametric data to compare between the three groups; P* ANOVA comparison between the three groups; P_1_ Fenofibrate versus curcumin; P_2_ Fenofibrate versus placebo; P_3_ Curcumin versus placebo

### Effect of fenofibrate versus curcumin on the studied parameters

The comparison between the three groups after three months of intervention was demonstrated in Table 2. Compared to their baseline data, weight, BMI, and waist circumference measurements were significantly decreased in the fenofibrate group after intervention (p < 0.05), whereas these measurements did not significantly change in the curcumin group (p > 0.05). Moreover, weight and BMI did not differ significantly between the three groups, whereas waist circumference measurements were significantly decreased in the fenofibrate group compared to the curcumin group (p < 0.05).

**Table 2:**
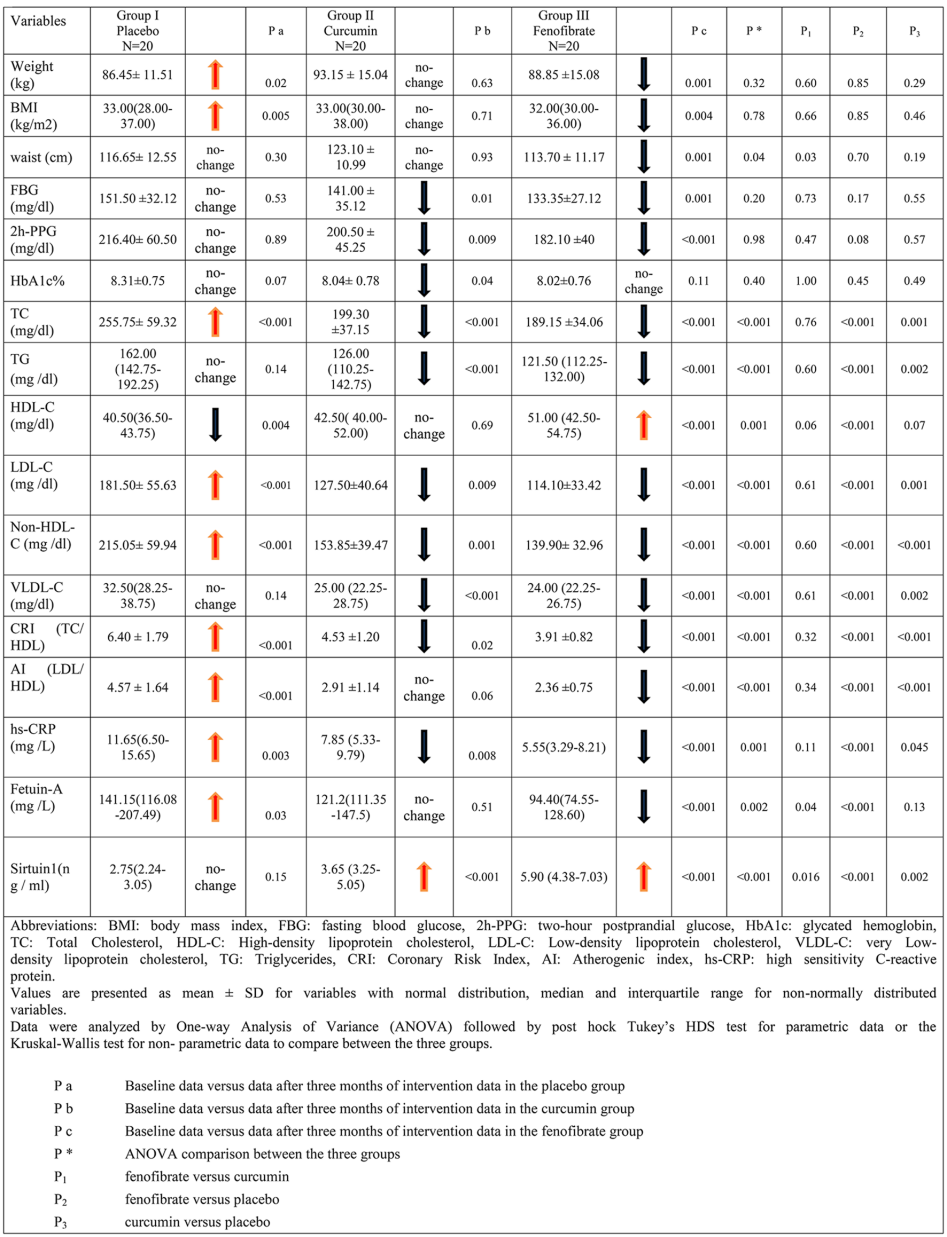
Comparison between the three studied groups after three months of intervention

FBG and 2 h-PPG levels were significantly decreased in the fenofibrate group (p ≤ 0.001) and the curcumin group (p < 0.05) after intervention compared with the baseline. HbA1c levels did not significantly change in the fenofibrate group (p > 0.05), while they decreased significantly in the curcumin group (p < 0.05). In the placebo group, FBG, 2 h-PPG, and HbA1c levels were not significantly changed (p > 0.05). The mean differences in FBG, 2 h-PPG, and HbA1c were not statistically significant between the studied groups (p > 0.05).

Regarding lipid profile, a significant decrease was observed in lipid panel measures (TC, TG, LDL-C, non-HDL-C, VLDL-C), and CRI, in the fenofibrate (p < 0.001) and curcumin (p < 0.05) groups compared with baseline. The fenofibrate group was the only group that showed a statistically significant elevation in HDL-C and a significant reduction in AI (p < 0.001) compared to baseline data. Significant reductions were found in lipid panel measures, CRI, and AI in the fenofibrate (p < 0.001) and curcumin (p < 0.05) groups compared with placebo after intervention. The fenofibrate group showed a statistically significant increase in HDL-C compared with the placebo group (p < 0.001).

Serum levels of hs-CRP were significantly decreased in the fenofibrate group (p < 0.001) and the curcumin group (p < 0.05). Fetuin-A levels significantly decreased in the fenofibrate group (p < 0.001), whereas they did not change in the curcumin group (p > 0.05). Sirtuin 1 serum levels were significantly increased in the fenofibrate and the curcumin groups (p < 0.001) at the end of the study period compared with baseline. Compared to the placebo group, the serum levels of hs-CRP were significantly decreased in the fenofibrate (p < 0.001) and curcumin (p < 0.05) groups and the serum level of sirtuin 1 was significantly increased in the fenofibrate (p < 0.001) and curcumin (p =  0.002) groups. In the same context, serum levels of fetuin-A were significantly decreased in the fenofibrate group compared with those in the curcumin group (p < 0.05) and the placebo group (p < 0.001). The sirtuin 1 level was significantly increased in the fenofibrate group compared to that in the curcumin group (p < 0.05).

**Fig. 2:**
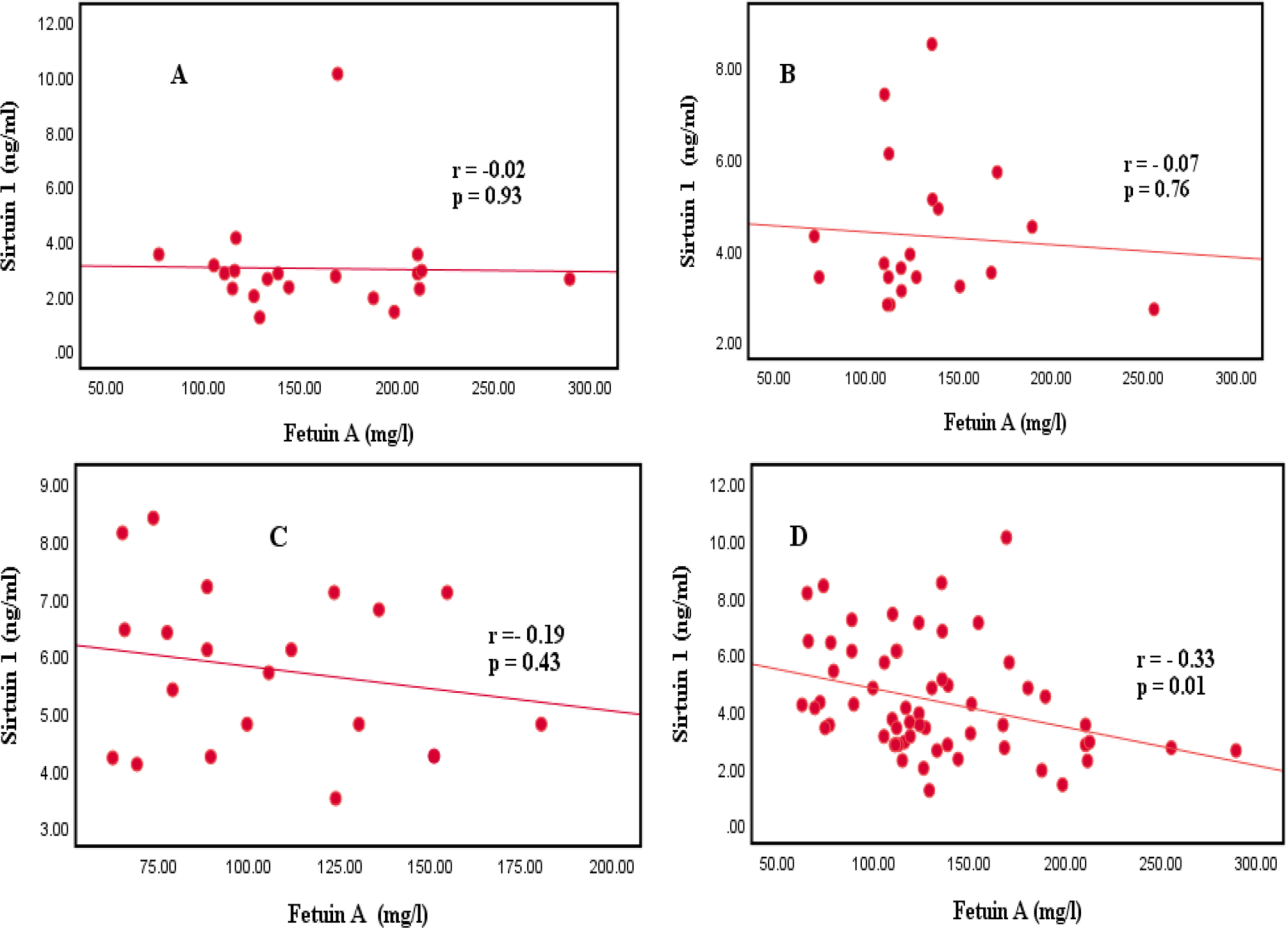
Correlation between levels of fetuin-A and sirtuin-1 after three months of treatment in the studied population and the correlation in each group. **A** Placebo group, **B** Curcumin group, **C** Fenofibrate group, **D** The three studied groups

Figure 2 shows Pearson’s correlation analysis. A significant negative correlation was observed between fetuin-A and sirtuin 1 level after three months of intervention in the three studied groups, statistically significant at p < 0.05. During the study, curcumin tablets were well tolerated, and no side effects were reported. Fenofibrate tablets were also tolerated; however, three out of 20 (0.15%) patients complained about nausea, but this complaint disappeared after two weeks of treatment. Liver function tests were performed for all patients who received fenofibrate, and there were no abnormal levels or severe problems with the drug.

## Discussion

In this study, we compare the effects of adding fenofibrate versus curcumin to glimepiride versus glimepiride alone on weight, glycemic status, lipid profile, hs-CRP, fetuin-A, and sirtuin 1 in patients with type 2 diabetes. Three months after the intervention, and compared to their baseline variables, the fenofibrate group showed weight reduction, decreased BMI, and reduced waist circumference measurements. These results agreed with the findings of a study on metabolic syndrome in obese patients, which showed that fenofibrate significantly decreased body weight, BMI, and waist circumference measurements [31]. Moreover, another study on rats reported that fenofibrate decreased food intake [32]. Also, a study in obese rats postulated that fenofibrate appears to stabilize weight primarily by its effect on liver metabolism [33]. In contrast, after three months of intervention, curcumin therapy showed a nonsignificant change in the measurements of weight, BMI, and waist circumference. These current results are in accordance with former findings that illustrated that curcumin does not significantly influence BMI [34]. Furthermore, another study concluded that curcumin does not improve body weight [35]. However, these findings disagreed with the results of a study on patients with nonalcoholic fatty liver disease (NAFLD), which reported that curcumin significantly reduced BMI [36]. Our results showed no statistically significant changes in weight and BMI between the three groups. However, waist circumference measurements were significantly different between the three groups. The fenofibrate group showed a significant decrease in waist circumference measurements compared to the curcumin group. These results were in accordance with Filippatos et al. [31], who reported that fenofibrate significantly decreased waist circumference.

Fenofibrate therapy resulted in a significant reduction in FBG and 2 h-PPG (p < 0.001) and a nonsignificant but clinically significant decrease in glycated hemoglobin. These findings are in accordance with the results of a meta-analysis of 22 randomized control trials, which showed that fenofibrate reduced fasting blood glucose significantly with no effect on glycated hemoglobin [37]. Similarly, a study done on type 2 diabetes patients with hypertriglyceridemia postulated that fenofibrate was effective in improving the glycemic pictures of the patients [38]. Consistently, Murthy et al. illustrated that in rats and rabbits, fenofibrate had a significant effect on glimepiride’s hypoglycemic activity [39]. Moreover, curcumin significantly reduced FBG, 2 h-PPG, and glycated hemoglobin after three months of treatment (p < 0.05). These results are consistent with the finding of a former study [40] that found serum glucose and glycated hemoglobin decreased in the case of curcuminoids in type 2 diabetes. Moreover, a systematic review of patients with diabetes illustrated that curcumin significantly reduced fasting blood glucose and glycated hemoglobin [16]. In the placebo group, the decrease in 2 h-PPG was not statistically significant and glimepiride therapy did not significantly change FBG and glycated hemoglobin (p > 0.05). These results contradicted the findings of a previous review [7] demonstrating that glimepiride regimens significantly reduced FPG, PPG, and HbA1c values. The effect of glimepiride is attributed to its mechanism of stimulating insulin release by acting on ATPase-dependent potassium channels in pancreatic cells, in addition to its extrapancreatic effects and cardiovascular effects (maintaining myocardial preconditioning) [7]. On the other hand, no statistically significant difference was observed between the three groups regarding FBD, 2 h-PPG, and HbA1c.

Both fenofibrate and curcumin showed significant reductions in TC, TG, LDL-C, non-HDL-C, and VLDL-C levels after three months and compared to baseline. These findings were in line with those of previous studies done on fenofibrate [8] and curcumin [17], indicating that fenofibrate and curcumin improve lipid profile. However, these beneficial effects in improving lipid profile were associated with an increase in HDL-C in the fenofibrate group only (p < 0.001) and no significant changes in HDL-C in the curcumin group. Our findings regarding HDL-C in the curcumin group are consistent with those of a systematic review and meta-analysis on patients with metabolic syndromes and related diseases to detect curcumin effects on glycemic status and lipid profiles, where no significant effects of curcumin on HDL-C levels were detected [41]. However, this systemic review and meta-analysis showed that curcumin had no effect on LDL-C, which disagreed with our results concerning LDL-C levels since our findings indicated a significant decrease in LDL-C levels with curcumin supplementation. On the contrary, Yung et al. found that curcumin significantly increased HDL-C; however, the study was conducted on patients with polycystic ovary syndrome [42]. The CRI was significantly decreased in the fenofibrate and curcumin groups, while the AI significantly decreased in the fenofibrate group and did not change in the curcumin group. The CRI and AI are considered strong markers for predicting atherosclerosis and coronary heart disease [43]. Hence, fenofibrate showed a better effect than curcumin in preventing cardiovascular diseases, and these results agree with those of many trials that established the importance of fenofibrate in decreasing cardiovascular events [44, 45]. Fenofibrate and curcumin significantly decreased TC, TG, CRI, and AI compared to placebo. These beneficial effects on lipid profiles associated with fenofibrate and curcumin compared to placebo emphasized the importance of medications used to treat hyperlipidemia associated with T2D to reduce the risk of cardiovascular diseases [46].

In the present study, hs-CRP serum levels were significantly reduced in the fenofibrate group (p < 0.001). These results were in agreement with those of Coban et al. [47], who reported that fenofibrate improves low-level chronic inflammation in patients who were obese with dyslipidemia by decreasing serum hs-CRP levels. Moreover, Wu et al. [48] demonstrated that fenofibrate significantly decreased hs-CRP. Our results showed that fetuin-A levels were reduced significantly in the fenofibrate group, which is in accordance with former findings [49]. Sirtuin 1 levels were significantly increased in the fenofibrate group and these findings are matched previously reported outcomes [49, 50].

In our study, curcumin significantly decreased hs-CRP after three months of treatment, and these results are consistent with those of another study that explained the beneficial effects of curcumin and its ability to decrease the inflammatory markers and decrease hs-CRP [17]. Moreover, a previous meta-analysis also showed that curcumin supplementations reduce CRP levels and hs-CRP concentrations [51]. Curcumin did not significantly improve levels of fetuin-A among patients with diabetes. Our findings were in accordance with those of another study done on NAFLD patients, where no significant difference was observed in fetuin-A serum levels between the curcumin group and placebo [52]. However, our results concerning fetuin-A levels in the curcumin group contradict those of two studies done on rats, which reported that curcumin decreased fetuin-A levels [53, 54]. In the curcumin group, sirtuin 1 levels were significantly increased (p < 0.001). These outcomes were in agreement with those of Yang et al. [55], who reported that curcumin pretreatment aids in activating SIRT1 signaling. Furthermore, Zendedel et al. [56] postulated that curcumin supplementation increased serum levels of sirtuin 1 and had antioxidant and anti-inflammatory properties.

The findings in our study concerning the inflammatory markers (fetuin-A, and sirtuin 1) showed a superior effect of fenofibrate compared to curcumin in decreasing fetuin-A and increasing sirtuin1. To our best knowledge this is the first study comparing the effect between fenofibrate and curcumin on the inflammatory markers (fetuin-A, and sirtuin 1). Fenofibrate acts as a lipid modifying agent through the activation of peroxisome proliferator-activated receptor-α. It is a fibric acid derivative used to treat adults with primary hypercholesterolemia, mixed dyslipidemia, and hypertriglyceridemia. Fenofibrate has pleiotropic effects and can lower fibrinogen, C-reactive protein, and uric acid levels [57], which could explain why it improves lipid profiles and inflammatory markers. Moreover, curcumin’s effect could be attributed to its unique chemical structure, which allows it to interact with a diverse range of molecular targets. The biological effects may include the inhibition of reactive oxygen species (ROS) production, which is essential, especially for diseases associated with oxidative stress [11]. Curcumin can affect glucose homeostasis and diabetic complications, and it improves insulin resistance, increases the release of adiponectin, reduces leptin levels, resistin, tumor necrosis factor-α, and interleukin- (IL-) 6 [58]. A negative correlation was found between fetuin-A and sirtuin 1 levels after three months of intervention in the studied patients. Since some studies found elevated levels of the fetuin-A present in diabetes, macrovascular complications, and late-stage diabetic nephropathy, the negative correlation could be attributed to the level of each inflammatory marker in the case of diabetes and related complications [23]. On the other hand, reduced levels of sirtuin 1 are associated with glucose intolerance, insulin resistance, and metabolic syndrome [59]. Inconsonance a recent study demonstrates that sirtuin 1 can predict worsened insulin resistance [57]. Furthermore, this relationship may explain the negative correlation between serum fetuin-A and sirtuin 1, as observed in our findings.

### Study limitations

The small sample size and short follow-up period are the primary limitations of this trial. So, additional large-scale trials and longer duration studies are required.

## Conclusions

To the best of our knowledge, our study is the first to compare the effects of adding fenofibrate versus curcumin on glimepiride therapy on weight, glycemic status, lipid profile, hs-CRP, fetuin-A, and sirtuin 1 in patients with type 2 diabetes. In summary, the current study showed the substantial benefit of adding fenofibrate compared to curcumin in patients with type II diabetes treated with glimepiride. Both drugs were effective in improving the glycemic picture, lipid profile, and inflammatory markers when added to glimepiride therapy. These findings confirmed their pleiotropic properties as an anti-inflammatory and cardioprotective agents. However, fenofibrate might have a superior effect in reducing waist circumference and inflammatory markers (reducing fetuin-A and increasing sirtuin 1). On the other hand, larger, well-designed, prospective clinical trials are still required to accurately detect the difference between curcumin versus fenofibrate effects in patients with type 2 diabetes.

## Data Availability

Data supporting the findings of this study are available upon request.
